# Responsive Hydrogels from Associative Block Copolymers: Physical Gelling through Polyion Complexation

**DOI:** 10.3390/gels3010003

**Published:** 2017-01-01

**Authors:** Christine M. Papadakis, Constantinos Tsitsilianis

**Affiliations:** 1 Fachgebiet Physik weicher Materie, Physik-Department, Technische Universität München, James-Franck-Str. 1, 85748 Garching, Germany; 2 Department of Chemical Engineering, University of Patras, 26504 Patras, Greece

**Keywords:** physical hydrogel, polyelectrolyte, pH-responsive, block polyampholyte, polyion association, ionic interactions, three-dimensional (3D) network

## Abstract

The present review article highlights a specific class of responsive polymer-based hydrogels which are formed through association of oppositely charged polyion segments. The underpinning temporary three-dimensional network is constituted of hydrophilic chains (either ionic or neutral) physically crosslinked by ion pair formation arising from intermolecular polyionic complexation of oppositely charged repeating units (polyacid/polybase ionic interactions). Two types of hydrogels are presented: (i) hydrogels formed by triblock copolymers bearing oppositely charged blocks (block copolyampholytes), forming self-assembled networks; and (ii) hydrogels formed by co-assembly of oppositely charged polyelectrolyte segments belonging to different macromolecules (either block copolymers or homopolyelectrolytes). Due to the weak nature of the involved polyions, these hydrogels respond to pH and are sensitive to the presence of salts. Discussing and evaluating their solution, rheological and structural properties in dependence on pH and ionic strength, it comes out that the hydrogel properties are tunable towards potential applications.

## 1. Introduction

In the last decades, hydrogels have attracted extraordinary attention from theoretical computational, experimental and application point of view, thanks to their valuable properties that are suitable for a large variety of applications [1,2,3,4,5]. Hydrogels are water-born soft materials based mainly on a three-dimensional (3D) network, formed either by small or large organic molecules, through hierarchical self-assembly and/or crosslinking procedures On the one hand, appropriate macromolecules for the 3D network creation constitute hydrophilic chains bearing functional pendant or end groups capable of undergoing crosslinking reactions (chemical gels). On the other hand, amphiphilic polymers, namely associative polymers [6,7], are the best-suited building elements for the formation of the so-called physical hydrogels. In this case, short hydrophobic sequences, attached to long hydrophilic chains, play the role of stickers which associate intermolecularly, through hydrophobic interactions, forming non-permanent reticulation nodes. Beyond these two initially appearing categories of hydrogels, new developments have emerged in the field, due to the enormous progress of macromolecular chemistry, encompassing supramolecular and “click” chemistry, opening new strategies for designing novel polymeric materials as innovative hydrogelators. For instance, well-designed functional block copolymers, of various topologies, have been involved for the fabrication of physical hydrogels and endowed them with novel functionalities. The so-formed hydrogels are referred also as self-assembling hydrogels [8].

The involved block copolymers are constituted of a hydrophilic major part which stabilizes the hydrogel. It can be either a neutral (in most of the cases) or an ionic chain, bearing a number of ionic groups along the chain. The associative part is responsible for the development of the secondary intermolecular interactions, namely hydrophobic, ionic, H-bonding, π–π stacking, host/guest, etc., which drives the macromolecules to self-assemble in water, creating the 3D network structure. Hydrophobic interactions, exerted from the hydrophobic stickers, are the most widely studied case in hydrogels. Depending on the strength of hydrophobicity, correlated with the exchange dynamics of the stickers from the reticulation nodes, dynamic or “frozen” hydrogels can be formed [9].

“Smart” hydrogels belong to a relatively novel class of hydrogels, arising mostly from the self-assembly of the so-called stimuli-responsive block copolymers, triggered by various stimuli such as temperature, pH, light, etc. [10,11,12,13,14]. The latter strategy endows the hydrogels with some particular properties like self-healing and injectability [15,16] allowing “in situ gelling” [17] that meets the requirements for some specific biomedical applications like tissue engineering [18] and controlled drug delivery [19]. For instance, the hydrophobic association can be triggered by a stimulus like temperature or pH when the stickers are hydrophilic sequences, exhibiting lower critical solution temperature (LCST) behavior or suitable p*K*_α_, respectively [20].

Beyond hydrophobic interactions, other non-covalent intermolecular secondary interactions have recently been used for designing “smart” hydrogelators. Among those, metal–ligand coordination has been utilized to crosslink macromolecules into 3D structures [21]. The so-formed physical gels, referred to as metallo-supramolecular polymer gels, are based on 3D networks where the linkages between the macromolecules are provided by reversible, labile metal–ligand coordination bonds. Recently, a supramolecular stimuli-responsive polymer gel was fabricated by well-designed heterotelechelic block copolymers, one extremity being ended by a short associating sticker and the other bearing a chelating ligand. Through the hydrophobic and coordination terminal moieties, the copolymer was hierarchically associated into a supramolecular network with tunable viscoelastic response and yield behavior [22]. An early review summarizes work up to 2006 [23].

Ionic interactions between oppositely charged repeating units (polyacid–polybase), located in the same or in different macromolecules, constitute another strategy to design “smart” hydrogels, and this is the topic of the present review. The hydrogel formation relies on the oppositely charged polyion association, and it is driven by ion pairing (ionic bonds) and the entropy gain due to the release of counterions and hydration water [24], leading to the formation of polyion complexes which form the physically reversible cross-links of the transient network, also named complex coacervate cores (CCCs) [25,26]. The involved interactions between the oppositely charged segments were referred, in most of the cases in the literature (also by us) as electrostatic, which seems inappropriate according to the thermodynamics of polyion association which is mainly entropic [27,28,29]. Herein, we use the term “ionic interactions” characterizing the interactions between oppositely charged groups (segments) that lead to electro-neutralization of the formed polyion complexes [24]. Depending on the environmental conditions, e.g., presence of salt, these interactions can lead to the formation of the so-called interpolyelectrolyte complexes, namely: (i) (dense) polyelectrolyte complexes or complex precipitates; and/or (ii) polyelectrolyte coacervates (hydrated domains), which are characterized by loose association and liquid like properties [30,31].

By using weak electrolyte repeating units, the so-formed hydrogels are strongly pH-responsive since pH affects the degree of ionization of the oppositely charged polyions and hence their extent of association, rendering them promising candidates for biomedical applications. Two types of systems, block copolymer polyampholytes and mixtures of two oppositely charged polyions (either in the form of triblock/pure polyelectrolyte or triblock/triblock), will be presented and discussed.

We note here that from the strictly rheological point of view, physical hydrogels are soft solids (elastic response), exhibiting very long relaxation times (i.e., very slow exchange dynamics) much higher than the experimental time, having, hence, immeasurable zero-shear viscosity and yield behavior. However, a broader definition of hydrogels, also referred to as free-standing gels (in the time of observation), comprise systems that feature viscoelasticity with measurable long relaxation times and high viscosities, has been used in the literature. In this article, the broader definition is adopted.

The paper is structured as follows. In the first section, we describe systems based on charge-driven self-assembly, afforded by one block copolymer polyampholyte. Afterwards, systems based on charge-driven co-assembly, comprising two oppositely charged macromolecules, are presented. In each case, the solution properties as well as the rheological and structural properties of the hydrogels are discussed. Finally, we summarize the findings and give an outlook.

## 2. Systems Based on Self-Assembly

The first system enabling self-assembly in aqueous environment was formed by asymmetric triblock copolymers having negatively charged short end blocks and a positively charged long middle block [32]. In addition to the usual possibilities to vary the network properties by variation of the block lengths, the choice of the nature of the polyelectrolyte (strong versus weak) controls the properties of the hydrogels. Moreover, the pH value (weak polyelectrolyte case) and the ionic strength have a strong influence. The system based on the triblock polyampholyte PAA-*b*-P2VP-*b*-PAA (APA) offers great variability because the degrees of ionization of both blocks are pH-dependent: poly(acrylic acid) (A) has a p*K*_a_ of 4.5 and poly(2-vinyl pyridine) (P) a p*K*_b_ of 5.0. Thus, varying pH alters the net charge and the anion/cation molar ratio along the polymer. Moreover, the uncharged monomers of both blocks are either hydrophobic or capable of developing H-bonding, and their association contributes to the charge-driven self-assembling behavior and the mechanical properties.

### 2.1. Dilute Solution Properties

**Figure 1:**
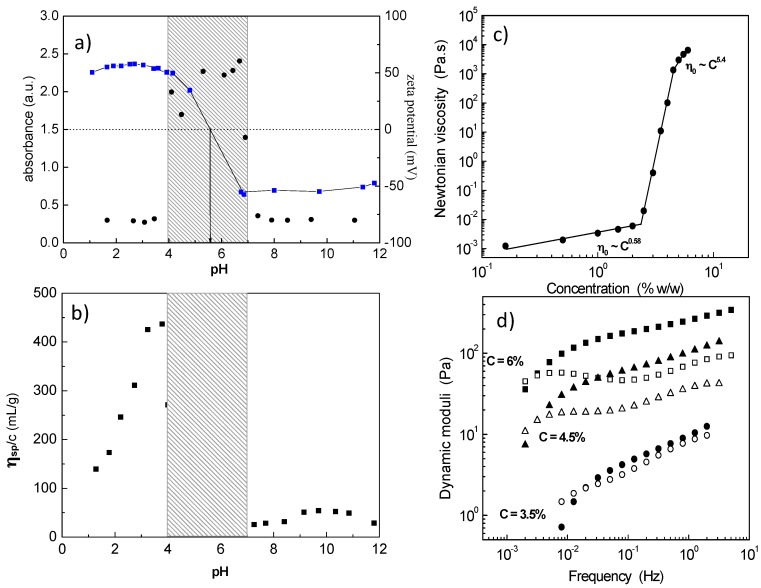
pH dependence of: (**a**) the optical absorbance (black symbols), the zeta potential (blue symbols); and (**b**) of the reduced viscosity, η_sp_/*c*, for a APA_1_ aqueous solution (0.2 wt %) at 25 °C. (**a**,**b**) Adapted with permission from [32]. Copyright 2003 American Chemical Society. (**c**) Zero-shear viscosity as a function of polymer concentration; (**d**) Storage modulus *G′* (closed symbols) and loss modulus *G*″ (open symbols) as a function of frequency at different polymer concentrations: circles (3.5 wt %), triangles (4.5 wt %), squares (6.0 wt %). Adapted with permission from [33]. Copyright 2004 American Chemical Society.

The intermolecular association of the PAA_134_-*b*-P2VP_628_-*b*-PAA_134_ (APA_1_) block polyampholyte towards a 3D network was revealed exploring salt-free dilute aqueous solutions by electrophoresis (zeta potential), turbidimetry (visible light) and capillary viscometry (reduced viscosity) as a function of pH (Figure 1a,b) [32]. The system exhibited three phases: (i) low pH, clear solution; (ii) intermediate pH, two phases, namely the isoelectric point (*iep*) region; and (iii) high pH, clear solution, where the polyampholyte has been transformed to an amphiphilic polyelectrolyte (charged A and hydrophobic P). At pH values just prior to the insoluble *iep* region, a maximum in the reduced viscosity indicated intermolecular association which leads to a 3D network by increasing concentration (Figure 1c) [33]. The percolation threshold was determined at 2.4 wt % polymer concentration, while above 4.5 wt %, a free-standing gel was observed. The hydrogel exhibited shear thinning properties and viscoelastic response with relaxation times of the order of hundreds of seconds (Figure 1d). The network formation was attributed to the ionic association among the protonated positively charged P and a limited number of deprotonated, negatively charged A.

Recent developments in this system encompass quaternization of the P block resulting in PAA-*b*-QP2VP-*b*-PAA (A(QP)A) with the strong cationic polyelectrolyte quaternized poly(2-vinyl pyridine) (QP) as the long middle block (absence of hydrophobic uncharged P units), which renders it soluble in the entire pH range (cationic P moieties always predominate). In addition, only the A end blocks exhibit a pH-dependent degree of ionization, facilitating the control of the charge imbalance (anion/cation molar ratio) which promotes better understanding of the system.

Parallel investigations of these two triblock polyampholytes (of the same block lengths) in dilute solutions (0.2 wt %) revealed that the quaternized version of the triblock polyampholyte, PAA_163_-*b*-QP2VP_1397_-*b*-PAA_163_ (A(QP)A_2_), stayed water-soluble in the entire pH region, which was attributed to its high net charge as was corroborated by the positive zeta potential for pH values between 1 and 13 [34]. In contrast, PAA_163_-*b*-P2VP_1397_-*b*-PAA_163_ (APA_2_) precipitated above pH 5, and its zeta potential changed sign from positive below pH 5, where the ionized P predominates, to negative above pH 7, where the deprotonated A segments prevail. Thus, both polyampholytes had a positive net charge in the low pH region, which was of interest for the formation of hydrogels.

### 2.2. Rheological Properties of the Hydrogels

**Figure 2:**
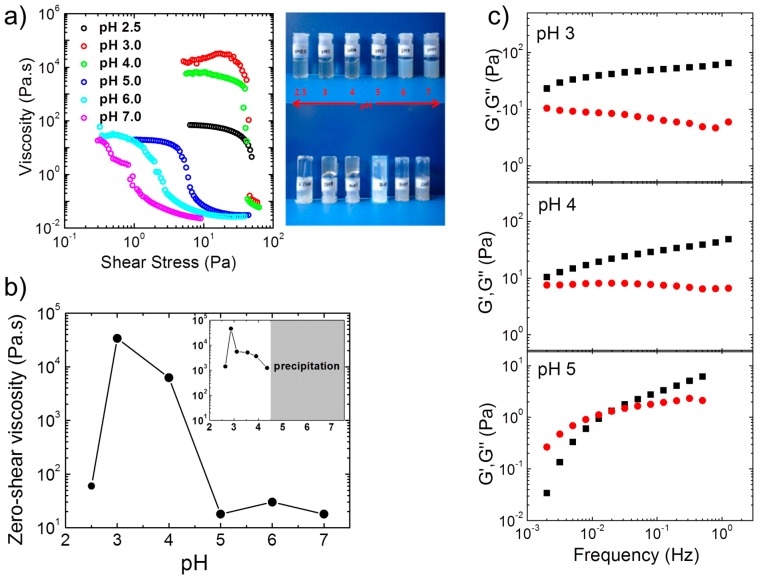
pH-dependent rheological properties of salt-free aqueous solutions of A(QP)A_2_. (**a**) **Left**: Apparent viscosity as a function of shear stress for A(QP)A_2_ at *c* = 4 wt % and various pH conditions; **right**: photos showing free-standing gels at pH 3 and 4, whereas solution behavior is observed at other pH values; (**b**) pH dependence of zero-shear viscosity, η_0_, at *c* = 4 wt % and its precursor APA_2_ at *c* = 1.2 wt % (**inset**); (**c**) Dynamic moduli, *G*′ (black symbols) and *G*″ (red symbols) versus frequency at different pH values of A(QP)A_2_ at *c* = 4 wt % at the pH values given in the graphs. Adapted with permission from [34]. Copyright 2014 American Chemical Society.

Steady-state shear viscosity measurements and tube inversion tests on more concentrated (4 wt %) aqueous solutions of A(QP)A_2_ at pH values between 2.5 and 7.0 revealed the formation of transparent, free-standing hydrogels (Figure 2a), particularly at pH 3 and 4. Gelation occurred in the same pH region as for the non-quaternized precursor (APA_2_), and both systems exhibit maximum value in zero shear viscosity close to pH 3 (Figure 2b). Thus, the driving force for the formation of 3D network is of ionic nature (polyion complexation), since, in the quaternized version, all P moieties of the central block are permanently charged, and hence hydrophobic interactions and H-bonding with A are negligible. The drastic effect of the charge imbalance was manifested in the marked decrease of the viscosity at pH 5 and 2.5, which demonstrated that the degree of ionization of the A end blocks, which are weak polyelectrolytes, is at the origin of the pH responsive behavior. This pH dependent behavior was confirmed in oscillatory measurements in the linear viscoelastic regime (Figure 2c). At pH 3 and 4, *G*′ was higher than *G*″ in the whole frequency range with relaxation times higher than 500 s, which confirmed the appearance of free-supporting hydrogels at these pH values. At pH 5, in contrast, viscoelastic behavior was observed with a terminal relaxation time of ca. 50 s. For the non-quaternized triblock polyampholyte APA_2_, the zero shear viscosity at 1.2 wt % was also maximum at pH 3, however, precipitation set in above pH 4 (inset of Figure 2b). For this polymer, the viscoelastic behavior appeared at lower pH: At pH 4.35, the terminal relaxation time already decreased to 13 s. Both observations confirmed the important role of the increasing hydrophobicity of the P block with pH.

**Figure 3:**
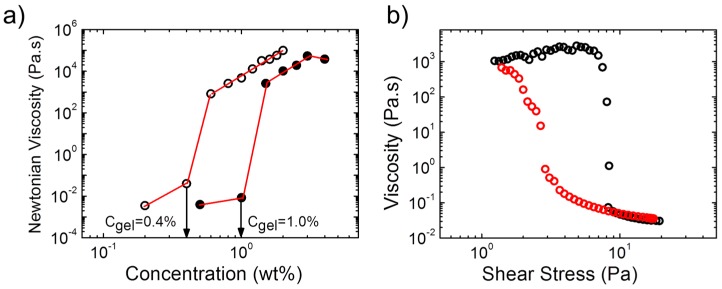
(**a**) Concentration dependence of the zero shear viscosity at the pH of maximum viscosity for A(QP)A_2_ (closed symbols) and APA_2_ (pH 2.9) (open symbols). Lines along the data guide the eyes and arrows indicate the gelation concentration; (**b**) Apparent viscosity versus shear stress of 1.6 wt % aqueous A(QP)A_2_ solution at pH 3: increasing stress (black circles) and decreasing stress (red circles). Adapted with permission from [34]. Copyright 2014 American Chemical Society.

The concentrations, at which gel formation sets in, were found to be very low for the two polymers and to differ slightly (Figure 3a): For A(QP)A_2_, the zero-shear viscosity increased by ca. 6 orders of magnitude between a polymer concentration of 1.0 wt % and 1.5 wt %, which pointed to the formation of a transient network above this concentration, critical gel concentration (*C*_gel_). For APA_2_, the same behavior was observed, however, *C*_gel_ was with ca. 0.4 wt % much lower than in the quaternized copolymer. Thus, *C*_gel_ also depends remarkably on the ratio of the opposite charges. We should also note that *C*_gel_ is remarkably lower than that of APA_1_ (Figure 3a), indicating the influence of the molecular features of the copolymer on the rheological properties and network structure. The nonlinear behavior of the hydrogel from A(QP)A_2_ was probed in a steady state shear experiment (Figure 3b). At a concentration just above *C*_gel_, shear thickening was observed prior a dramatic shear thinning, marked by a drop of the viscosity of five orders of magnitude at a relatively low stress (ca. 8 Pa), implying easy disruption of the network structure. Remarkable hysteresis was observed, pointing to the slow structure recovery, in accordance with the long relaxation time observed by oscillatory measurements (Figure 2c).

Thus, rheological characterization established that both, ionic and hydrophobic (for the non-quaternized polyampholyte) interactions are of importance for the formation of a 3D network. These are accessible by altering pH and thus the degrees of ionization of the oppositely charged moieties.

### 2.3. Structural Properties of the Hydrogels in Dependence on pH and Ionic Strength

**Figure 4:**
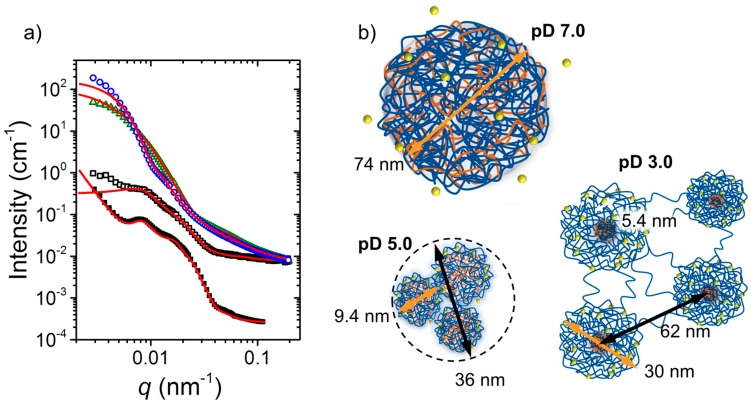
SANS results from A(QP)A_2_ and APA_2_. Data from [34]. (**a**) Scattered intensity, *I*(*q*), as a function of the momentum transfer, *q*, of solutions of A(QP)A_2_ at pD 7.0 (open blue circles), pD 5.0 (open green triangles) and pD 3.0 (open black squares), and APA_2_ at pD 3.0 (closed black squares, shifted vertically for clarity). All scattering curves were measured at a concentration of 4 wt % and at 26 °C. The red lines are the model fits; (**b**) Corresponding nanostructures at the pH values given. Blue lines: QP, orange lines: A, yellow circles: counter ions. Average length scales are given. Adapted with permission from [34]. Copyright 2014 American Chemical Society.

At a concentration safely above *C*_gel_, namely 4 wt % (Figure 3a), a pronounced effect of the pH value on the mechanical properties was observed (Figure 2). At this concentration in heavy water, D_2_O, the structural characteristics also showed gross differences, as found using small-angle neutron scattering (SANS). The SANS curves from the quaternized polyampholyte A(QP)A_2_ at pD 7.0, 5.0, and 3.0 as well as the one from APA_2_ at pD 3.0 are compiled in Figure 4a. As evident from their very different shapes, the morphology depends strongly on the charge imbalance (controlled by pH). The curves at pD 3.0 from A(QP)A_2_ and APA_2_ have similar shape, but the features are shifted along the momentum transfer, i.e., the length scales involved differ. Structural models were fitted to describe the morphology and to extract structural parameters.

The scattering curve of A(QP)A_2_ at pD 7.0, where the charge imbalance is minimum (degree of ionization of the A block ca. 80%) and extended ionic interactions take place, was successfully modeled with large spherical particles (radius ~37 nm) together with concentration fluctuations (for details see [34]). These particles had a loose internal structure with an inner correlation length of ~2.6 nm. They were composed of complexed negatively charged groups of the A end blocks and of positively charged groups of the long QP middle block together with about 14 wt % of water (coacervate type structures). The ratio of positively and negatively charged chains within the particle was imbalanced (due to asymmetric blocks), and the excess positively charged QP moieties stabilized the particles. This morphology—charged, unconnected, microgel-like associates of mesoscopic size, as sketched in Figure 4b—is in accordance with the low viscosity at pH 7.0 (Figure 2b).

At pD 5.0, the charge imbalance on the chain was higher due to the smaller fraction of negatively charged A units (~50%). The SANS curve (Figure 4a) was successfully modeled by small spherical core-shell particles having an average core radius of 3.4 nm and a shell thickness of 1.3 nm. The cores consist again of CCCs from negatively charged A segments and positively charged QP segments. The smaller size than the one at pD 7.0 is due to the increasing charge imbalance, resulting in smaller polyion complexes. The shell contains the remaining QP groups. These associates formed larger micellar clusters, containing ca. 50 small micelles. From the viscoelastic behavior described above, it was concluded that these clusters are loosely connected (Figure 4b) and may be disrupted by shear forces.

At pD 3.0, the charge imbalance is maximum with ca. 25% of the A segments being charged (note that the apparent p*K*_a_ of A is lowered due to the presence of QP [34]). The SANS curve was fitted using a model including the form factor of polydisperse spherical core-shell particles which are correlated with each other. The particles were found to have an average core radius of ~3 nm and a (rather high) shell thickness of ~12 nm. The average distance between the particle cores was ~60 nm. Thus, small cores were surrounded by a shell of (possibly backfolding) QP blocks and connected with each other by QP bridges. This became possible since less QP segments were involved in the formation of the crosslinked domains than at the higher pD values. At pD 3.0, the sample thus forms a 3D network (Figure 4b) in accordance with the observed gel-like behavior.

The SANS curve of the non-quaternized triblock polyampholyte APA_2_ at 4 wt % in D_2_O pD 3.0 looked similar to the one of A(QP)A_2_, albeit shifted to lower values of the momentum transfer, *q*, and with more pronounced features. Again, spherical core-shell particles were found, having an average core radius of ~4 nm, which is larger than in A(QP)A_2_, probably because of the overall higher amount of uncharged, hydrophobic P segments, contributing to the core size augmentation. Accordingly, the shell thickness was found to be smaller than in A(QP)A_2_, namely only ~6 nm. The higher cross-link functionality is in accordance with the higher zero-shear viscosity observed in the non-quaternized hydrogel (Figure 3a). The hard-sphere radius was higher in APA_2_ (~36 nm in comparison with ~31 nm in A(QP)A_2_). Thus, the bridges between the crosslinked cores are longer, resulting in a higher connectivity, in accordance with the lower *C*_gel_ value for APA_2_.

The results from the systematic study of the self-assembled, quaternized and non-quaternized triblock polyampholyte system showed that the overall degree of ionization and the charge imbalance together with the hydrophobicity of the uncharged segments result in strongly pH dependent morphologies and, consequently, in vastly different rheological properties.

### 2.4. Influence of Ionic Strength

The above-described system was also found to be sensitive to ionic strength because the addition of salt reduces the charge density of both type of blocks [35]. It was found that the addition of salt may alter both, the stability of the complex coacervate cores and the bridging ability of the middle blocks. This became evident by comparing the effect of NaCl on two systems: (a) a hydrogel from (PAA_109_-*b*-P2VP_819_-*b*-PAA_109_) APA_3_ which was investigated at pD 3.0; and (b) a viscoelastic liquid formed by PAA_109_-*b*-QP2VP_819_-*b*-PAA_109_ (A(QP)A_3_) which was investigated at pD 5.0, both at a concentration of 3 wt %.

**Figure 5:**
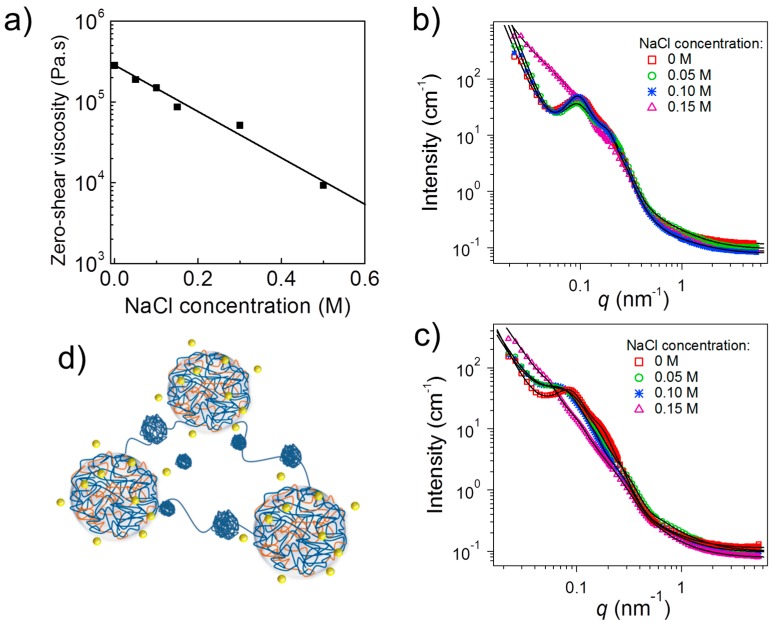
Effect of NaCl on the self-assembled hydrogels from A(QP)A_3_ and APA_3_. (**a**) Zero shear viscosity, *η_0_*, as a function of NaCl concentration of 3 wt % APA_3_ aqueous solutions at pH 3.0; (**b,c**) SANS curves of solutions of APA_3_ at pD 3.0 (**b**) and A(QP)A_3_ at at pD 5.0 (**c**), both at 3 wt % in D_2_O and at 26 °C for different NaCl concentrations (symbols) together with the model curves (lines); and (**d**) sketch of the morphology at a NaCl concentration of 0.15 M. Adapted with permission from [35]. Copyright 2015 American Chemical Society.

The zero-shear viscosity of APA_3_ at pH 3.0, extracted from creep measurements, diminished continuously with increasing NaCl concentration (Figure 5a). Starting from a rather high value, which reflects the stiffness of the hydrogel, in salt-free conditions, the viscosity decreased strongly with ionic strength. The ion-pairing dissociation induced by salt addition, weakens the integrity of the physical ionic crosslinks, facilitating therefore the exchange of the A chain-ends from their junctions, decreasing therefore the terminal relaxation times. The stability of these junctions, are affected by the NaCl ions, which are able to penetrate them and to break ionic bonds (through counterion exchange) between oppositely charged A and P blocks, thus leading to their partial disintegration and a softer gel. Due to the high polymer concentration, the number of salt ions was not sufficient, though, to break all polyion pairs, and, therefore, the elastic character of the hydrogels was preserved even at 0.5 M NaCl [35].

The viscosity changes upon salt addition were accompanied by morphological changes, as evidenced by SANS (Figure 5b). For the salt-free solution of APA_3_ at pD 3 as well as the ones at 0.05 M and 0.10 M, the scattering curves had a shape characteristic of a network formed by interconnected coacervate cores (cf. Figure 4a). Only at a salt concentration of 0.15 M, the shape strongly differed. At NaCl concentrations up to 0.10 M, coacervate domains having radii of ca. 8 nm were present (Figure 5d). The hard-sphere radius was ca. 30 nm. Thus, in this range of NaCl concentrations, SANS shows that there are no dramatic changes in the network architecture, but rather a redistribution of chains between the complexes.

At 0.15 M NaCl, the same structural model could be fitted. The average radius of the coacervate domains was ~17 nm, thus larger than that at lower NaCl concentrations. This was attributed to the increased hydrophobicity of the coacervate domains and to the screening of the charges of the P blocks, and that some of these uncharged P segments joined the coacervate domains. Moreover, additional small spheres were present, having an average radius of ~7 nm which were attributed to small globules of uncharged P (Figure 5d). Moreover, increased scattering at small momentum transfers indicated that smaller and looser aggregates of coacervate domains were present at 0.15 M NaCl, in contrast to the larger aggregates giving rise to a steep increase of intensity with decreasing momentum transfer at 0–0.10 M NaCl (Figure 5b). The addition of salt thus caused a breakup of the large aggregates which is in accordance with the decrease of the zero-shear viscosity discussed above.

**Figure 6:**
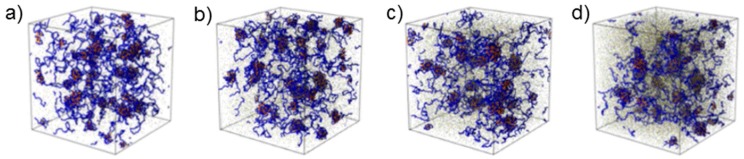
Snapshots from computer simulations of polyampholyte solutions PAA_20_-*b*-QP2VP_172_-*b*-PAA_20_ (A(QP)A_4_) at a polymer concentration of 2.2 wt % in salt-free solution (**a**); and at salt concentrations of: 0.153 M (**b**); 0.305 M (**c**); and 0.61 M (**d**). A and QP monomers are shown in orange and blue color, respectively. Reprinted with permission from [35]. Copyright 2015 American Chemical Society.

The A(QP)A system at pD 5.0 was strongly charged—the QP block was nearly fully charged and the fraction of charged A segments was ca. 55%. Thus, this system offered the possibility to investigate the role of ionic effects upon addition of salt which were expected to dominate over hydrophobic effects due to non-charged segments. The SANS curves again revealed network formation at low NaCl concentrations (Figure 5c), but—in contrast to the APA_3_ system at pD 3.0—changes were already apparent at 0.05 M NaCl. Model fitting revealed an average radius of the coacervate domains of ~7 nm in the salt-free state with an average hard-sphere radius of ~34 nm. Their radius grew steadily to ~19 nm at 0.15 M NaCl, which was attributed to the fact that the presence of small amounts of salt may enhance complex formation by weakening ionic interactions, favoring more accessible polymer conformations, and enabling chain rearrangement [36]. Computer simulations confirmed the growth of coacervate domains upon increasing ionic strength (Figure 6) [34]. Moreover, QP globules having an average radius of ~8 nm were again detected. The hard-sphere radius follows the same tendency as the radius of the complex coacervate domains.

Comparing the two systems, which were studied experimentally, reveals that the hydrogel formed by the non-quaternized APA_3_ at pD 3.0 is less sensitive to ionic strength than the ones from A(QP)A_3_ at pD 5.0. In the former system, the impact of screening by the small ions is not as pronounced as in the latter system because of the higher fraction of non-ionized segments. The hydrophobic interactions between non-ionized P segments hamper the effect of the salt addition. In the quaternized system, in contrast, strong effects of the salt on the network structure are observed, because ionic interactions play a dominant role in the highly charged system.

## 3. Systems Based on Co-Assembly

### 3.1. Co-Assembly of Triblock Copolymers Having Charged Endblocks and Oppositely Charged Homopolymers

Charge-driven co-assembly is based on the co-dissolution of oppositely charged polymers. These may form multiresponsive reversible gels which are sensitive not only to changes in temperature and concentration but also to ionic strength, cationic/anionic composition and, if weak polyelectrolytes are used, pH value. Such a system was designed by Lemmers et al. [37,38,39] who investigated the co-assembly of a triblock copolymer with negatively charged end blocks and a neutral, hydrophilic middle block with a chemically different, positively charged homopolymer. The hydrophilic middle blocks prevented phase separation but led instead to microphase separation. At low polymer concentrations, flower-like micelles with CCCs, arising from the polyion complexation of the negatively charged end blocks and the positively charged homopolymers and loops of the middle blocks forming the shell were observed. At higher concentrations, the micelles were bridged by the middle blocks, resulting in the formation of a transient micellar network.

**Figure 7:**
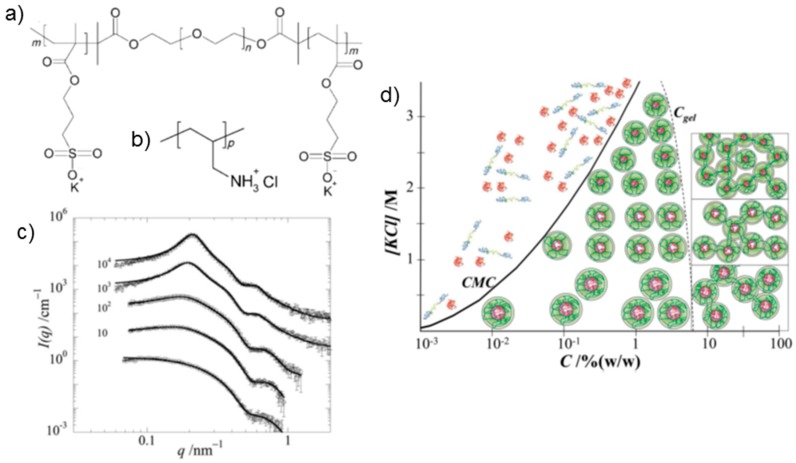
Co-assembled system formed by a triblock copolymer with negatively charged end blocks and: a neutral middle block (SES) (**a**); and AH homopolymer (**b**); (**c**) Small-angle X-ray scattering (SAXS) data for gels from SES and AH at 0.4 M KCl at 20 °C. The polymer concentrations are (from bottom to top) 1%, 4%, 6%, 12% and 20%. Lines are model fits. The curves are shifted vertically as indicated in the graph; (**d**) Schematic morphology diagram for gels from SES and AH in dependence on polymer concentration, *C*, and salt concentration [KCl]. Reproduced with permission from [38]. Copyright 2011 Royal Society of Chemistry.

The polymers under study were the triblock copolymer PSPMA_28_-*b*-PEO_210_-*b*-PSPMA_28_ (SES) (Figure 7a) and the homopolymer poly(allylamine hydrochloride) (AH) (Figure 7b). The appropriate mixing ratio was determined using titration dynamic light scattering on dilute solutions (ca. 1 g·L^−1^ in a 0.2 M KCl aqueous solution). At the composition where the scattering intensity was maximum, the triblock copolymers and the homopolymers formed micelles with a hydrodynamic radius of ca. 20 nm [37]. This occurred at the charge composition variable $f^{+}=\left[+\right]/\left(\left[+\right]+\left[-\right]\right)\approx 0.5$ (charge stoichiometry) where [+] and [−] denote the concentrations of positive and negative charges, respectively.

At higher polymer concentrations, the viscosity increases by several orders of magnitude between 5 wt % and 20 wt %, and the critical gel concentration *C*_gel_ was determined at 4 wt % [37]. Small-angle X-ray scattering (SAXS) at concentrations of 1–16 wt % revealed spherical particles having a radius of gyration of ca. 8 nm and a distance between them of ca. 30 nm [37]. Addition of KCl led to a decrease of both, the intensity of scattered light and the viscosity. Thus, the almost solid-like gel was transformed into a water-like fluid by adding KCl salt. The gels were also found to be pH responsive because the AH homopolymer is a weak polyelectrolyte. Increasing the pH of a dilute solution of micelles resulted in the decrease in the number of micelles above pH 8. In a gel formed at 18 wt % and 0.4 M KCl, the viscosity decreased drastically upon addition of KOH.

In a later study [38], the same polymers, but having slightly different molar masses, were investigated. A detailed SAXS investigation was carried out in a wide concentration range at 0.4 M (Figure 7c) and 1.0 M KCl. At the lowest polymer concentration, 1 wt %, nearly uncorrelated micelles were observed and could be described as homogeneous spheres having an average radius of 8 nm. With increasing concentration, the spheres became more strongly correlated, as evidenced from the more pronounced peak in the SAXS curves (Figure 7c). The sphere radius resulting from model fitting was nearly independent on concentration. The hard-sphere radius, obtained from the structure factor in the model function, did not depend on concentration either, which may be counterintuitive. However, the volume fraction of the spheres increased nearly linearly with polymer concentration up to 15 wt %, then it leveled off. From the resulting number density of micelles, the authors calculated an average center-to-center distance of the spheres, *D*. A decrease with increasing polymer concentration following the expected relation $D\propto C^{-1/3}$ was found. The aggregation number of the spheres was found to be ca. 110 at a KCl concentration of 0.4 M and to decrease linearly with [KCl].

Moreover, the mechanical properties were investigated [38]. In frequency sweeps, viscoelastic behavior was demonstrated for a 20 wt % polymer concentration at 0.4 M KCl. The plateau modulus was found to increase sharply between 8 wt % and 10 wt % which the authors attributed to an increasing number of bridges. Based on the structural and rheological characterization, the authors proposed a morphology diagram of the charge-driven, co-assembled system in dependence on polymer concentration and KCl concentration (Figure 7d). The critical micelle concentration (CMC), i.e., the concentration above which (flower-like) micelles form, increased with salt concentration. At high polymer concentration, the micelles were found to pack more closely and to be strongly bridged. The co-assembled charge-driven system thus offers great variability in terms of composition and responsivity to pH, ionic strength and temperature.

**Figure 8:**
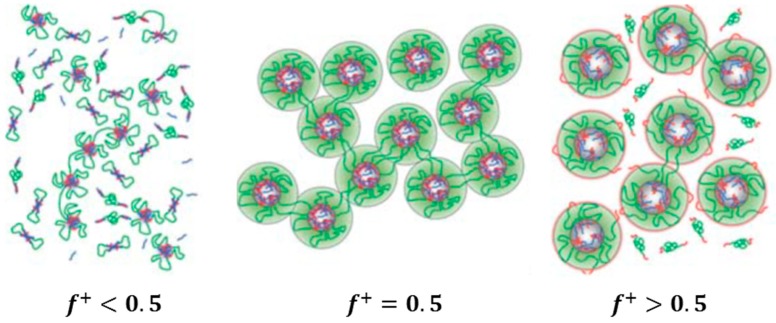
Schematics of the morphologies of solutions from SES and AH for excess negative charge, charge stoichiometry and excess positive charge for the concentrated regime. Adapted with permission from [39]. Copyright 2012 Royal Society of Chemistry.

In a later investigation, the authors investigated the effect of charge composition, $f^{+}$, while keeping the overall polymer concentration constant [39]. For $f^{+}<0.5$, excess negative charges persisted, leading to small, negatively charged aggregates (radius 5–10 nm) from negatively charged homopolymers and positively charged triblock copolymers, called “soluble complexes” (Figure 8). The viscosity was relatively low. At charge stoichiometry, $f^{+}=0.5$, the flowerlike micelles (radius 15–20 nm) were interconnected and tightly packed. At excess positive charge, $f^{+}>0.5$, the micellar size decreased, and a number of dangling positive end blocks stabilized the micelles, maintaining electroneutral complex cores. The authors suggested that bridge formation between these positively charged particles is suppressed, being at the origin of the relatively low viscosity.

**Figure 9:**
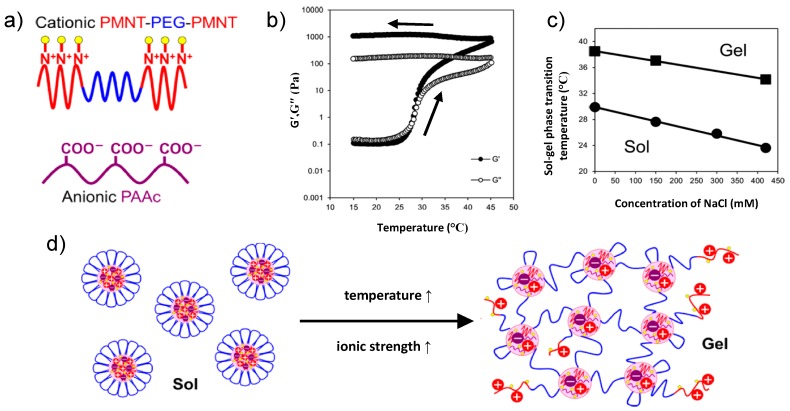
(**a**) Schematics of the polymers MEM and A; (**b**) storage modulus G′ and loss modulus G″ of polyion complexes flower micelles (55 mg/mL, r=1:1, 150 mM NaCl, pH 6.2, 550 mM phosphate buffer) with increasing temperature and decreasing temperature, as indicated by the arrows; (**c**) ionic strength dependence (55 mg/mL, r=1:1 , pH 6.2): concentration of phosphate buffer 330 mM (closed squares) and 550 mM (closed circles); and (**d**) schematics of the solution of the polyion complexes micelles and the irreversible gel formation upon increasing temperature and ionic strength. Adapted with permission from [40]. Copyright 2015 American Chemical Society.

Ishii et al. designed a system which is responsive to both, temperature and ionic strength and therefore qualifies as an injectable hydrogel for medical purposes [40]. Triblock copolymers featuring a water-soluble poly(ethylene glycol) (EG) middle block and two cationic poly[4-(2,2,6,6-tetramethylpiperidine-*N*-oxyl)-aminomethylstyrene] (M) end blocks were codissolved with anionic poly(acrylic acid) (A) homopolymers. PMNT-*b*-PEG-*b*-PMNT (M(EG)M) and A were mixed at several molar ratios in a phosphate buffer solution (Figure 9). At low concentrations, they formed polyion complexe flower-like micelles at room temperature. Heating a micellar solution having a molar ratio r=1:1 (the molar ratio was defined as the ratio of the molar unit of cationic amine groups of MEM and the molar unit of the anionic carboxyl groups of A and a concentration of 55 mg/mL) resulted in a steep increase of the storage and the loss modulus at 27.6 °C. Interestingly, for r=1:1 and 1:2, the high moduli persisted when cooling back from 45 °C to 15 °C, i.e., the gel formation was irreversible. The authors attributed the gel formation to the formation of ionic cross-links between the cationic MEM and the anionic A, namely by destruction of the flower-like structure and not by their aggregation. This is corroborated by the high modulus in the gel state (>1000 Pa). At low temperatures, the gel formation is prevented by the EG shell of the micelles. Gel formation occurred at concentrations as low as 2 wt %. For r=2:1, the gel formation was found to be reversible. The sol-gel transition temperature decreased upon addition of NaCl, the system is thus responsive to ionic strength. The authors suggested the use of the co-assembled hydrogel as injectable hydrogels: While the micellar solutions at room temperature have low viscosity and can be injected easily into the body, they form gels in the body because of an increase in both, temperature and ionic strength. Since a certain ionic strength is necessary, gelation will not happen in the catheter, but only in the body. It was shown that the system can also incorporate charged macromolecules and therefore may be used as a local delivery carrier of charged drugs.

### 3.2. Co-Assembly of Triblock Copolymers Having Oppositely Charged Endblocks

Several investigations have addressed responsive, co-assembled hydrogels formed by symmetrical triblock copolymers having the same hydrophilic middle block and oppositely charged end blocks [41,42]. Theoretical work [43] addressed the influence of the interaction parameters of the various segments with each other and with the solvents.

**Figure 10:**
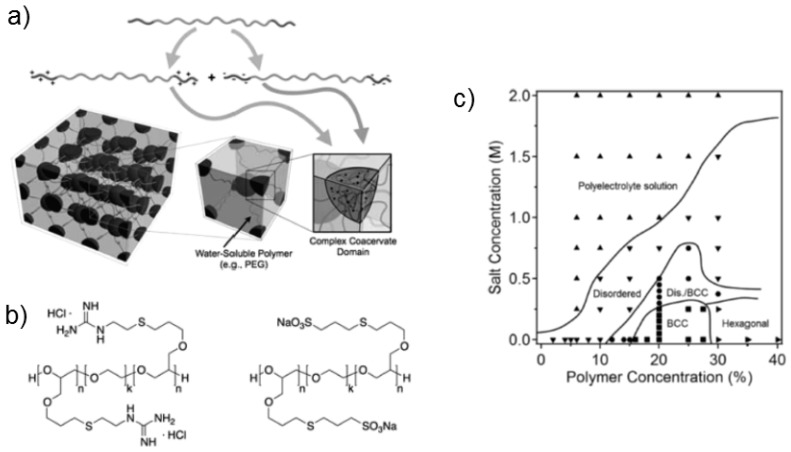
(**a**) Co-assembled system formed by charged triblock copolymers, which were synthesized from the same precursor having a hydrophilic middle block and uncharged end blocks. Schematics of co-assembly of the triblock copolymers having oppositely charged end blocks; (**b**) chemical structures of the charged triblock copolymers having guadinium and sulfonate groups; and (**c**) phase diagram of mixtures of these triblock copolymers in dependence on polymer and NaCl concentration. BCC stands for body-centered cubic. Reprinted with permission from [42]. Copyright 2013 American Chemical Society.

Hunt et al. [41] and Krogstad et al. [42] designed high performance hydrogels by exploiting the complex coacervate formation of oppositely charged end blocks of two triblock copolymers which have the same long and hydrophilic middle block (Figure 10a). These are both water-soluble, facilitating gel preparation by mixing. The coacervate domains formed by the end blocks are sensitive to pH and ionic strength, and the gels are thus responsive materials. The charged triblock copolymers were synthesized from a common uncharged precursor triblock copolymer, namely PAGE-*b*-PEG-*b*-PAGE ((GE)(EG)(GE)). The alkene groups in the poly(allyl glycidyl ether) (GE) end blocks were used to introduce ionic groups, namely sulfonate, carboxylate, ammonium or guanidinium. At this, the molar mass of the EG middle block was varied between 10 and 35 kg/mol and the number of charged groups in the end blocks between 22 and 53 per end block. Following this concept, charge imbalance could be avoided.

Mixing triblock copolymers having carboxylate and guadinium groups at 10 wt % resulted in transparent and mechanically robust hydrogels. In dynamic mechanical measurements, the shear and the loss modulus showed a crossover at 15.8 rad/s, i.e., they were viscoelastic. Hydrogels were also formed upon mixing of triblock copolymers with sulfonate and guadinium groups. These were more elastic with a longer relaxation time. When the number of charged groups was sufficiently high, the coacervate domains formed a body-centered cubic lattice, as evidenced using SAXS. The modular approach thus resulted in a versatile system which allows for tuning of the properties.

In a later work, the same group systematically investigated hydrogels formed by mixtures of triblock copolymers with sulfonate and guadinium groups (Figure 10b, [42]). They focused on the effects of polymer concentration and salt (NaCl) concentration as well as pH and stoichiometry of the charged moieties on the mechanical properties and the morphologies. The block lengths were chosen at 31 for the end blocks and 455 for the middle block. Using dynamic mechanical spectroscopy and SAXS, a phase diagram was mapped out (Figure 10c). Increasing the polymer concentration or decreasing the salt concentration resulted in more ordered morphologies. At low salt concentration and intermediate polymer concentrations, a body-centered cubic structure was observed, which transformed into a hexagonal structure at high polymer concentration.

In comparison with the system described by Lemmers et al. [37,38,39] described above, the authors concluded that the higher amount of bridges in the triblock copolymer mixtures promoted the formation of ordered morphologies which had not been observed in the triblock copolymer/homopolymer system. The system presented [42] was proposed for the use as membranes, for injectable drug delivery or as tissue growth scaffolds.

**Figure 11:**
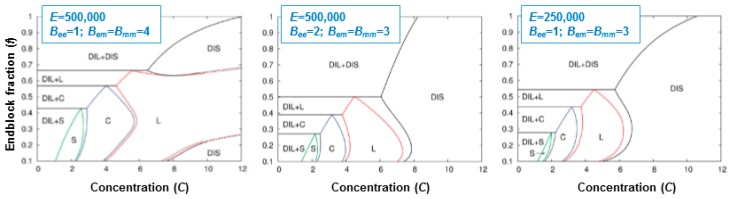
Phase diagrams of mixtures of triblock copolymers with a hydrophilic middle block and oppositely charged end blocks, calculated using the embedded fluctuation model. The morphologies are given in dependence on polymer number concentration and on the endblock fraction for different sets of parameters. *E* is the electrostatic strength parameter. DIS denotes a polymer rich homogeneous phase, DIL a nearly pure water phase, L lamellae, C hexagonally packed cylinders and S cubically packed spheres (body-centered or face-centered). Adapted with permission from [43]. Copyright 2015 Royal Society of Chemistry.

Theoretical work validated by computer simulations elucidated the role of various interaction parameters on the phase behavior of mixtures of triblock copolymers with oppositely charged end blocks [43]. The experimentally observed behavior (Figure 10c) could be qualitatively reproduced. Phase diagrams were presented in dependence on polymer concentration and endblock fraction (Figure 11). The following interaction parameters were considered: *B*_em_, which describes the strength of the (solvent-mediated) repulsive interaction between the middle block and the endblocks; *B*_mm_, which is a measure of the solvent quality for the middle blocks; and *B*_ee_, which is a measure of the endblock solubility in the solvent (higher values of *B*_mm_ and *B*_ee_ mean better solubility of the middle or the endblock, respectively).

The phase diagrams showed that ordered morphologies could be obtained for low endblock fractions. In this regime, the morphology may be altered by hydration or dehydration. Comparing the phase diagrams obtained for different parameter sets, it was found that increasing *B*_em_ or *B*_mm_ promoted microphase separation. Increasing the value of *B*_ee_, i.e., improving the end block solubility in the solvent, reduces the microphase-separated regions in the phase diagram. Decreasing the electrostatic strength parameter, *E*, decreased the driving force for microphase separation. This could be achieved by reducing the charge density on the endblocks. These results allow identifying the polymer architecture needed to obtain a desired morphology and to alter this morphology, e.g., by hydration or dehydration.

Cui et al. designed biodegradable hydrogels by co-assembly of triblock copolymers having oppositely charged polypeptides as endblocks and water-soluble EG as the middle block [44]. These hydrogels from PGA-*b*-PEG-*b*-PGA (G(EG)G, where PGA stands for poly(l-glutamic acid)) and PLL-*b*-PEG-*b*-PLL (L(EG)L, where PLL stands for poly(l-lysine)) could be reversibly assembled and disassembled through a change of pH. In more detail, when equimolar solutions of the triblock copolymers in phosphate-buffered saline (PBS) buffer were mixed, gels formed within a few seconds even at polymer concentrations as low as 3–5 wt %. Since both types of endblocks are weak polyelectrolytes, reversible gelation occurs at intermediate pH values only, whereas the gels disassembled at pH < 3 or pH > 11. In contrast to other systems [42], also described above, the addition of NaCl at 1 M led to an increase of the storage modulus of the hydrogels. The authors ascribed this phenomenon to the hydrophobic interaction of the polypeptides which maintained the stability of the coacervate domains, despite the decreasing polyionic interactions. The authors demonstrated furthermore the controlled release of the charged protein bovine serum albumin, the cell viability and the in vivo gel formation and maintenance in rats.

## 4. Summary and Outlook

The aim of this mini review is to highlight a particular class of physically crosslinked hydrogels, arising from the intermolecular association of hydrophilic triblock copolymers bearing polyion sequences. The hydrogel formation relies on the ionic interactions between the oppositely charged repeating units that lead to the formation of hydrated polyion complexes (complex coacervate nanodomains) which constitute the cross-links of the formed transient network. The driving force of the intermolecular association of the participating oppositely charged polyions in the aqueous media is mainly entropic due to ion pairing and the release of the counterions along with the water molecules.

Two systems are distinguished, depending on the polymer constituents involved in the network formation: (i) self-assembly of a highly asymmetric triblock polyampholyte (bearing cationic and anionic blocks); and (ii) co-assembly of mixtures of triblock polyelectrolytes (having polyionic end blocks) with oppositely charged polyions. In the first system, the remarkable charge asymmetry (i.e., a high charge imbalance) favors the network formation and its mechanical response. On the contrary, in the second system, a charge stoichiometry (charge balance) is needed for the best mechanical performance of the gel. In both cases, the charge ratio of the oppositely charged blocks can be tuned at will through controlled polymerization methods available for the synthesis of the copolymers (macromolecular engineering). A worthy of mention difference between the two systems is that the bridging chains between the crosslinked polyionic complexes of the network are ionic for the first one (arising from the excess part of the larger ionic middle block) and neutral for the second one (arising from the middle block of the copolymer). This might be the reason why the percolation concentration is considerably lower in the first system. Moreover, the length of the bridging chain in the second case is defined at will from the degree of polymerization of the middle block resulting in well-defined network structures.

Another classification can also be seen in the co-assembly systems, depending on the employed type of the polyions: (i) mixtures of a triblock copolymer with ionic end blocks and an oppositely charged homopolymer; and (ii) mixtures of two triblock copolymers with the same neutral central block and oppositely charged end blocks of the same degree of polymerization. In the latter system, equal concentrations of the different triblock copolymers provide exact charge stoichiometry. Moreover, ordered 3D structures can be achieved at elevated concentrations.

By using weak electrolyte repeat units in the copolymers, the hydrogel formation depends strongly on the pH of the medium, in all cases, since it affects the degree of ionization of the polyelectrolyte blocks and, in turn, the charge imbalance of the system. Provided that the network formation is based on ionic complexations, the presence of salt is critical as well. Thus, two external stimuli (pH, ionic strength) will affect the network structure and the mechanical response of the hydrogels, rendering them stimuli responsive.

It is well known that the pH responsive hydrogels are good candidates as injectable carriers of therapeutic means in biomedical applications. A novel property provided by these new polyion complex-based hydrogels is that the coacervate cores of the network are hydrated. This may, for certain drugs, favor their encapsulation with respect to the hydrophobically associated systems. Inspired by the unique, controlled and responsive properties of the hydrogels, demonstrated herein, novel hydrogel systems can be designed, meeting the requirements for ideal injectable hydrogels, exhibiting biocompatibility and biodegradability, as well as proper elasticity and rapid self-healing. Theoretical modeling and computer simulations may help in identifying relevant molecular architectures and conditions. Some recent examples towards this direction have already been presented in this review.

## Abbreviations

3D	three-dimensional
A	poly(acrylic acid), PAA
AH	poly(allylamine hydrochlorid)
APA	PAA-*b*-P2VP-*b*-PAA
APA_1_	PAA_134_-*b*-P2VP_628_-*b*-PAA_134_
APA_2_	PAA_163_-*b*-P2VP_1397_-*b*-PAA_163_
APA_3_	PAA_109_-*b*-P2VP_819_-*b*-PAA_109_
A(QP)A	PAA-*b*-QP2VP-*b*-PAA
A(QP)A_2_	PAA_163_-*b*-QP2VP_1397_-*b*-PAA_163_
A(QP)A_3_	PAA_109_-*b*-QP2VP_819_-*b*-PAA_109_
A(QP)A_4_	PAA_20_-*b*-QP2VP_172_-*b*-PAA_20_
CMC	critical micelle concentration
CCC	complex coacervate core
*C*_gel_	critical gel concentration
E	poly(ethylene oxide), PEO
EG	poly(ethylene glycol), PEG
G	poly(l-glutamic acid), PGA
GE	poly(allyl glycidyl ether), PAGE
(GE)(EG)(GE)	PAGE-*b*-PEG-*b*-PAGE
G(EG)G	PGA-*b*-PEG-*b*-PGA
*iep*	isoelectric point
L	poly(l-lysine), PLL
LCST	lower critical solution temperature
L(EG)L	PLL-*b*-PEG-*b*-PLL
M	poly[4-(2,2,6,6-tetramethylpiperidine-N-oxyl)-aminomethylstyrene], PMNT
M(EG)M	PMNT-*b*-PEG-*b*-PMNT
P	poly(2-vinyl pyridine), P2VP
PBS	phosphate-buffered saline
QP	quaternized poly(2-vinyl pyridine), QP2VP
S	poly(3-sulfopropylmethacrylic acid), PSPMA
SANS	small-angle neutron scattering
SAXS	small-angle X-ray scattering
SES	PSPMA_28_-*b*-PEO_210_-*b*-PSPMA_28_
